# Reliability of televisits for patients with mild relapsing–remitting multiple sclerosis in the COVID-19 era

**DOI:** 10.1007/s10072-022-05868-5

**Published:** 2022-01-11

**Authors:** Simona Toscano, Francesco Patti, Clara Grazia Chisari, Sebastiano Arena, Chiara Finocchiaro, Carmela Elita Schillaci, Mario Zappia

**Affiliations:** 1 Department “G.F. Ingrassia”, Section of Neurosciences, Neurology Clinic, University of Catania, Via Santa Sofia 78, 95123 Catania, Italy; 2 Department of Economics and Business, University of Catania, 95129 Catania, Italy

**Keywords:** Multiple sclerosis, Telemedicine, EDSS, Neurological examination, e-health, COVID-19

## Abstract

**Background:**

Evidence of the cost-effectiveness of telemedicine (TM) for the management of Multiple Sclerosis (MS) has been provided recently. However, some doubts persist about the accuracy of neurological examinations performed remotely.

**Objectives:**

This study investigated the reliability of neurological evaluations performed through TM in mild MS patients as compared with standard in-person visits.

**Methods:**

In total, 76 patients with relapsing–remitting MS and Expanded Disability Status Scale (EDSS) ≤ 3.5 were consecutively recruited. Of them, 40 patients (52.6%) accepted to undergo both in-person and TM evaluations with independent examiners within 48 h. We alternatively asked patients to assure or not the presence of a caregiver during TM visits. A satisfaction questionnaire was administered to all participants.

**Results:**

The inter-rater agreement attributed by two independent neurologists during TM visit was high (κ > 0.80) for EDSS and Functional Systems (FS) scores. Moderate agreement between TM and in-person evaluations emerged for pyramidal (κ = 0.57; p < 0.001), brainstem (κ = 0.57; p < 0.001), bowel and bladder (κ = 0.54; p < 0.001) and sensory (κ = 0.51; p < 0.001) FS scores, higher in patients providing the support of a caregiver. A good reliability was reported for EDSS scores computed during remote and in-person visits (ICC = 0.83; 95% CI 0.70–0.91; p < 0.001).

**Conclusions:**

Despite the complexity of neurological examination, TM could be useful in monitoring MS patients with low disability.

**Supplementary Information:**

The online version contains supplementary material available at 10.1007/s10072-022-05868-5.

## Introduction

In recent years, the digital revolution and the near-universal spread of Internet have changed the doctor–patient communication and the way clinicians take care of patients. Patients use the Internet seeking for health information in all stages of their condition, from symptom onset to long-term management, often logging on to blogs and social media which are easy to use, inexpensive and interactive [1]. On the other hand, several platforms based on direct or indirect interactions with a medical team have been set up in the last few years and the medical community is faced with a new modality of doctor–patient relationship.

According to the European Commission, telemedicine (TM) aims to use information and communication technology to remotely provide healthcare services to patients [2], falling under the comprehensive concept of e-health [3]. Particularly, during the Coronavirus Disease 2019 (COVID-19) pandemic, the need to find an alternative way to manage chronic diseases, despite travel restrictions and bans on gatherings, has emerged. In this context, TM has allowed clinicians and healthcare providers to share information with patients outside the hospital or the outpatient setting [4], to guide neurorehabilitation for disabled patients [5] and to perform visits through videoconferencing. Preliminary results provided evidence of the cost-effectiveness and feasibility of TM and of patients’ and physicians’ satisfaction with the new modality [6, 7]. In patients suffering from multiple sclerosis (MS), TM has already provided positive results [5, 8–10], including the recent evidence of a near-comparability between neurological examinations performed during teleconsultations and in-person visits [10].

In this background, we conducted a pilot study to investigate the reliability of neurological evaluations performed through TM compared with in-person visits in patients with MS. In order to test the reliability of TM evaluations under less demanding conditions, we only included patients whose previous Expanded Disability Status Scale (EDSS) was lower or equal than 3.5, thus only reflecting the involvement of Functional Systems (FS) and not being influenced by gait impairment. This was also motivated by the greater ease of recruiting patients with mild MS, who access to MS centers to a larger extent due to treatment-related reasons and account for 50–55% of the total MS population [11]. We further investigated the reasons for patients’ hesitancy or willingness to undergo remote evaluations and their satisfaction with the use of TM.

## Methods

### Participants

In this pilot single-center study we consecutively recruited 76 patients with MS referring to the Neurology Clinic of the University Hospital “G. Rodolico” of Catania. A diagnosis of relapsing–remitting MS (RRMS) according to McDonald’s diagnostic criteria 2017, an EDSS ≤ 3.5, no history of clinical relapses and/or steroid treatment during the previous month was considered mandatory for the enrolment. To achieve this goal, we consecutively asked patients meeting the inclusion criteria to undergo both an evaluation through TM and an in-person visit. We alternatively asked patients to assure (PwC) or not (PwoC) the presence of a caregiver during remote visits. Demographic and clinical data were extracted from a computerized database, iMed© (Merck Serono SA; Geneva), routinely used to store real-time clinical information during outpatient visits.

The study was approved by our local ethical committee. All patients accepting to enter the study signed written informed consent.

### Conduction of in-person and remote visits

All patients underwent an in-person visit at the MS Centre of the Neurology Clinic in the University Hospital G. Rodolico, in Catania. The in-person evaluations, performed according to clinical practice, were conducted by a first neurologist, who attributed the EDSS and FS scores (pyramidal, cerebellar, brainstem, sensory, visual, bowel and bladder, cerebral). All patients received a unique link through email to join remote visits, within 48 h prior or after the in-person visit. Each remote visit with a single patient was performed as an audio–visual call arranged on Skype platform by using a corporate account for the MS Centre. The remote visit was conducted by a second neurologist, who guided the patient in the execution of the required tasks (see Appendix 1) and calculated the EDSS and FS scores. A third neurologist joined the remote visit from a different location without taking direct actions and independently attributed the FS and the EDSS score based on the same neurological examination. All neurologists worked at the MS Centre of the Neurology Clinic in the University Hospital G. Rodolico of Catania, and all were blinded to the scores attributed by other examiners and did not have access to the clinical data and reports of previous evaluations.

### Telemedicine Satisfaction Survey

Within 48 h after the completion of remote visits, all patients were invited to answer an online satisfaction survey about TM visit, which was adapted from an online questionnaire on TM [12] (Appendix 2). After 5 days, reminders were e-mailed to all participants. The survey consisted of 20 questions, exploring doctor–patient communication (Q1-Q3), technical difficulties in the use of TM (Q4-Q9), time and money saving for patients (Q10-Q11), emotional and practical aspects of TM (Q13-Q17), satisfaction of patients and caregivers (Q18-Q20). The survey included dichotomous (Q1-Q2; Q4; Q8-Q11; Q16-Q18), multiple-choice (Q5-Q7) and interval scale questions (Q3; Q12-Q15; Q19-Q20) requiring a given score from 1 to 10 (1 = at all; 10 = very much).

### Statistical analysis

Normally distributed continuous variables were reported as mean ± standard deviation (SD) and compared between groups with Student’s t test. Median and range were provided for ordinal data, compared between groups with Wilcoxon test. Cohen’s kappa coefficient (κ) was used to evaluate the inter-rater reliability between the EDSS and FS scores reported by the first and the second neurologists during the in-person and the TM visits, both for the entire study population and separately for PwC and PwoC. κ coefficient was also reported for the remote inter-rater agreement between EDSS and FS scores attributed by the second and the third neurologists. The strength of κ was considered as follows: < 0 = poor; 0–0.19 = slight; 0.20–0.39 = fair; 0.40–0.59 = moderate; 0.60–0.80 = substantial; 0.80–1.00 = almost perfect [13]. The difference between EDSS scores and FS scores computed by the first and the second examiners was also reported as ΔEDSS and ΔFS. The intraclass correlation coefficient (ICC) was reported to test the absolute agreement for single measures between EDSS and FS scores attributed by two different examiners during TM and in-person visits. Values of reliability were considered as follows: poor (less than 0.5), moderate (0.5–0.75), good (0.75–0.90), excellent (greater than 0.90) [14].

Binary logistic regression analyses were performed by considering the in-person and remote inter-rater agreement and the willingness to undergo TM evaluations as dependent dichotomous variables.

Demographic characteristics (sex, age, educational attainment, residence), clinical ones (disease duration, age at MS onset, EDSS, current treatment, therapeutic switches) and the presence of a caregiver during TM visit were considered as independent variables.

We used SPSS© (IBM Corp. IBM SPSS Statistics for Windows, Version 25.0) for statistical analysis. We considered a p value < 0.05 as significant for all tests.

## Results

### Study population characteristics

The characteristics of the study population are shown in Table 1. Out of 76 recruited patients, 40 (52.6%) accepted to undergo both remote and in-person visits, while 36 (47.4%) did not. Patients who accepted to undergo TM visits exhibited a mean age at study entry of 38.4 ± 9.2 years, 31 (77.5%) were resident outside the municipality of Catania, and 21 (52.5%) graduated from high school (52.5%). The majority of patients accepting TM (21; 52.5%) were treated with second-line DMDs and exhibited a median EDSS score of 1.0 (0.0–3.5).

**Table 1:** Demographic and clinical characteristics of the study population

	Patients accepting TM	Patients refusing TM	p value^a^
All patients N	40	36	-
Age at study entry Years (mean ± SD)	38.4 ± 9.2	43.6 ± 13.1	0.05
Sex N (%)			0.60
Males	8 (20.0)	9 (25.0)	
Females	32 (80.0)	27 (75.0)	
Educational attainment N (%)			0.20
Primary school or less	0 (0)	2 (5.6)	
Middle school	6 (15.0)	10 (27.8)	
High school	21 (52.5)	14 (38.9)	
Degree	13 (32.5)	10 (27.8)	
Residence N (%)			0.99
Municipality of Catania	9 (22.5)	8 (22.2)	
Other municipalities in the Province of Catania	21 (52.5)	19 (52.8)	
Other provinces	10 (25.0)	9 (25.0)	
Disease duration Months (mean ± SD)	109.2 ± 88.1	144.5 ± 103.0	0.11
Age at MS onset Years (mean ± SD)	29.3 ± 9.3	31.5 ± 10.5	0.32
EDSS Median (range)	1.0 (0.0–3.5)	1.5 (0.0–3.5)	0.28
Current treatment N (%)			0.09
First-line DMDs	19 (47.5%)	24 (66.7%)	
Second-line DMDs	21 (52.5%)	12 (33.3%)	
Therapeutic switches N (%)			0.15
No switches	20 (50.0%)	19 (52.8%)	
One	9 (22.5%)	13 (36.1%)	
Two or more	11 (27.5%)	4 (11.1%)	

TM = telemedicine; EDSS = Expanded Disability Status Scale; MS = multiple sclerosis; N = number; DMDs = disease-modifying drugs; SD = standard deviation

^a^Student’s t test was used to compare means between groups for continuous variables. Chi-squared test was used to detect significant differences between the expected and the observed frequencies between groups

The age at study entry was significantly lower in patients who accepted to enter the study (p = 0.05), while other demographic variables were not different between groups.

The unwillingness to undergo TM visits was the reason for refusal for 20 out of 36 patients (55.6%), while 5 (13.9%) were not inclined to undergo in-person visit due to COVID-19 pandemic, 3 (8.3%) had some difficulties in using technologies, and 2 (5.6%) did not succeed in initiating TM visit. Additionally, 6 patients (16.7%) refused because of the impossibility to assure the presence of a caregiver.

### Remote and in-person inter-rater agreement

The EDSS and FS scores attributed during in-person and TM visits are reported in Table 2. The visit duration was shorter in remote (13.6 ± 4.0 min) compared with in-person evaluations (19.1 ± 2.6 min) (p < 0.001). No meaningful differences emerged in the assignment of EDSS and FS scores, except for visual FS score, underestimated during TM visits and characterized by a certain degree of disagreement (κ = 0.13; p = 0.17). EDSS scores showed a fair inter-rater agreement, while the inter-rater reliability was moderate for pyramidal, brainstem, bowel and bladder and sensory FS scores. A fair agreement was reported for cerebral and cerebellar FS scores. Particularly, pyramidal, brainstem and bowel and bladder FS scores were overestimated during TM visits, while others were underestimated (Table 2).

**Table 2:** Comparison between EDSS and FS scores attributed by two different neurologists during in-person and remote visit in the study population (40 patients)

	First examiner, in-person visit	Second examiner, TM visit	Delta^a^	Cohen’s κ (p value)
EDSS score Median (range)	1.0 (0.0–6.0)	1.0 (0.0–5.5)	-0.05	0.30 (< 0.001)
Pyramidal FS score Median (range)	1 (0–3)	1 (0–2)	-0.03	0.57 (< 0.001)
Cerebellar FS score Median (range)	0 (0–2)	0 (0–2)	0.1	0.28 (0.02)
Brainstem FS score Median (range)	0 (0–3)	0 (0–3)	-0.13	0.57 (< 0.001)
Sensory FS score Median (range)	0 (0–3)	0 (0–2)	0.03	0.51 (< 0.001)
Visual FS score Median (range)	0 (0–5)	0 (0–1)	0.38	0.13 (0.17)
Bowel and bladder FS score Median (range)	0 (0–2)	0 (0–3)	-0.1	0.54 (< 0.001)
Cerebral FS score Median (range)	0 (0–2)	0 (0–2)	-0.1	0.39 (0.001)

TM = Telemedicine; EDSS = Expanded Disability Status Scale; FS = functional system

^a^For each score, delta was calculated as the difference between scores attributed during the in-person examination and the remote, respectively. Positive values represent an underestimation of scores attributed during TM visit compared with in-person one. Negative values represent an overestimation of scores attributed during TM visit compared with in-person one

A good reliability was reported for EDSS scores (ICC = 0.83; 95% CI 0.70–0.91; p < 0.001). A moderate reliability was assessed for pyramidal (ICC = 0.67; 95% CI 0.45–0.81; p < 0.001), bowel and bladder (ICC = 0.66; 95% CI 0.45–0.80; p < 0.001), brainstem (ICC = 0.65; 95% CI 0.43–0.80; p < 0.001), sensory (ICC = 0.62; 95% CI 0.38–0.78; p < 0.001) and cerebral (ICC = 0.59; 95% CI 0.35–0.76; p < 0.001) FS scores. The reliability was poor for cerebellar FS score (ICC = 0.42; 95% CI 0.13–0.64; p < 0.001), while not significant for visual FS score.

When comparing PwC and PwoC, a shorter duration of remote visit emerged in the first group (11.9 ± 2.7 vs 15.3 ± 4.5 min; p = 0.006). The remote and in-person inter-rater agreement, expressed as κ coefficient, was higher in PwC for all scores but pyramidal FS (0.48 vs 0.63, respectively) (Table 3).

**Table 3:** Inter-rater agreement in the attribution of EDSS and FS scores between in-person and remote visits in the study population, PwoC and PwC

	Patients without caregiver (N = 20)	Patients with caregiver (N = 20)
EDSS score κ (p value)	0.14 (0.09)	0.41 (0.001)
Pyramidal FS score κ (p value)	0.63 (< 0.001)	0.48 (0.002)
Cerebellar FS score κ (p value)	0.17 (0.30)	0.32 (0.14)
Brainstem FS score κ (p value)	0.44 (0.001)	1.00 (< 0.001)
Sensory FS score κ (p value)	0.28 (0.10)	1.00 (< 0.001)
Visual FS score κ (p value)	-0.07 (0.44)	1.00 (< 0.001)
Bowel and bladder FS score κ (p value)	0.42 (0.01)	0.77 (< 0.001)
Cerebral FS score κ (p value)	0.35 (0.02)	0.44 (0.05)

EDSS = Expanded Disability Status Scale; FS = functional system

### Remote inter-rater agreement

No differences emerged in the attribution of EDSS and FS scores by two examiners during TM visits. Accordingly, the inter-rater reliability for all FS scores was high, with the lowest agreement reported for the cerebellar FS (κ = 0.81; p < 0.001). The agreement detected in the EDSS score assignment was significantly high (κ = 0.88; p < 0.001).

### Patients’ satisfaction questionnaire

Out of 40 participants, 30 patients (75%) answered the satisfaction questionnaire as requested (Appendix 2). All patients felt at ease at communicating their major problems and understood the indications provided by the neurologist (Q1-Q2). On a scale of 1 to 10, the remote visit was considered considerably accurate (8.1 ± 2.0) (Q3), engaging (7.8 ± 2.1) (Q13-Q14) and practical (8.6 ± 1.5) (Q12-Q15).

Only 3 out of 30 patients (10%) found some difficulties in performing TM visit, in activating and maintaining a good connection (Q4-Q6). They experienced difficulties in hearing or seeing the examiner, as well as in being heard, and reported a loss of connection or some problems with the audio-to-video synchronization (Q7-Q9).

**Fig. 1:**
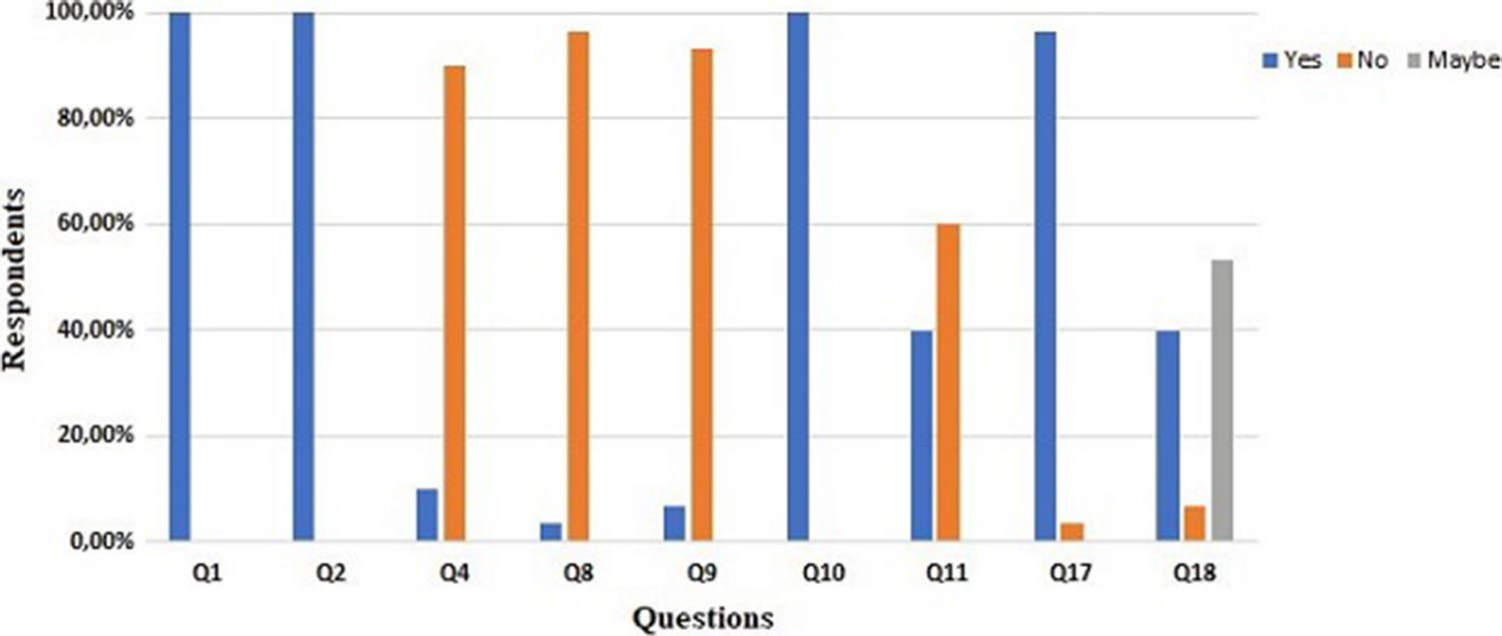
Answers to the Telemedicine Satisfaction Survey, *Telemedicine Satisfaction Survey is reported in Appendix 2. Only categorical questions and corresponding answers are reported.

All patients affirmed that the remote visit was performed at the scheduled time (Q10), while 12 patients (40%) declared to have saved money (Q11). The setting chosen by patients was sufficiently adequate to perform the remote visit and was easily found by all of them (Q16-Q17) (Fig. 1).

Comprehensively, 12 patients out of 30 (40%) declared their willingness to undergo further visits through TM, 16 (53.3%) had some doubts, and 2 (6.7%) reported to be not inclined to use TM again (Q18).

**Fig. 2:**
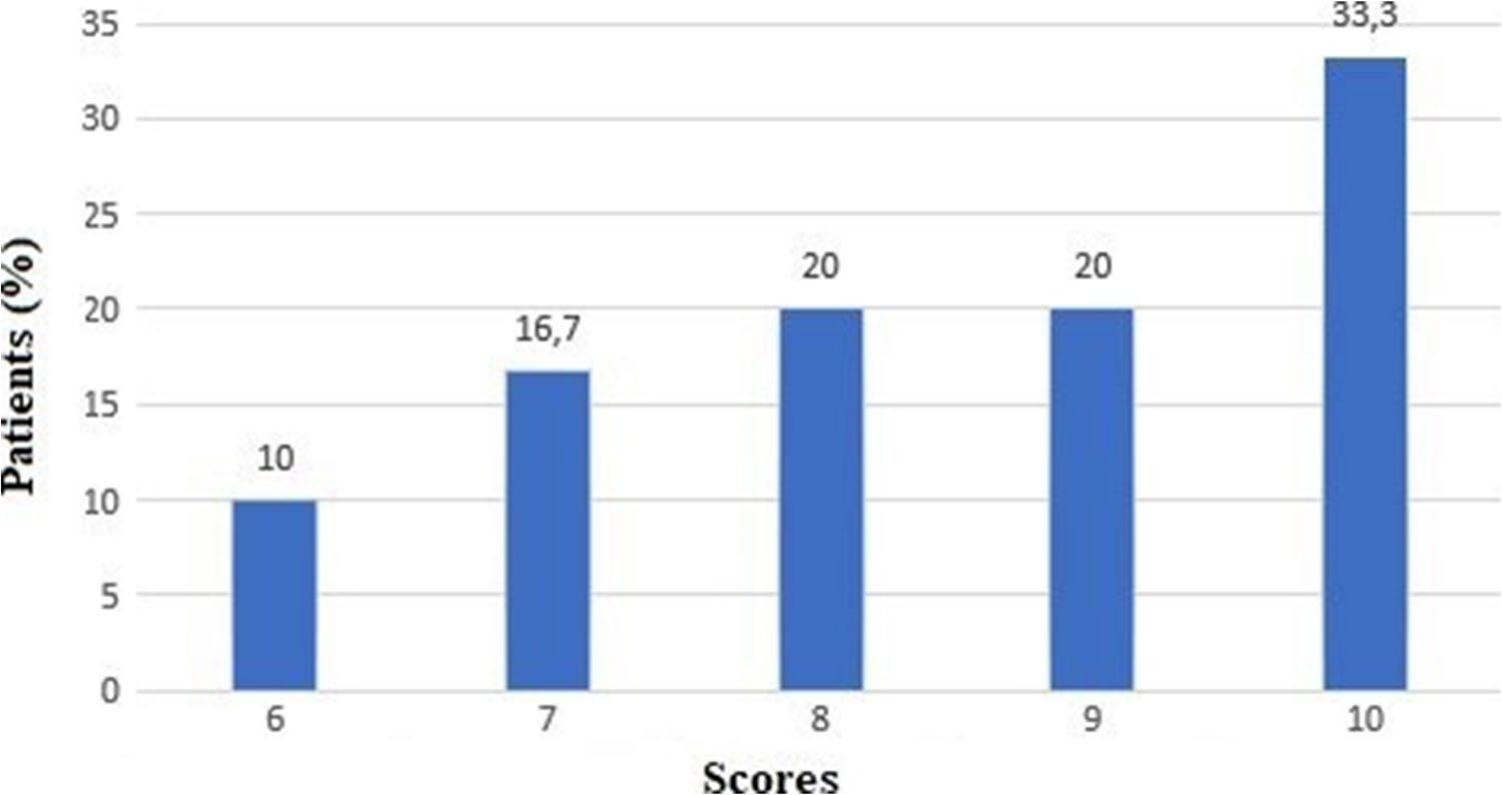
Satisfaction scores on a 1-to-10 scale given by patients (N = 30) to visit performed through TM (Q19), *No patients gave a score lower than 6 on a 1-to-10 scale. Telemedicine Satisfaction Survey is reported in Appendix 2.

**Fig. 3:**
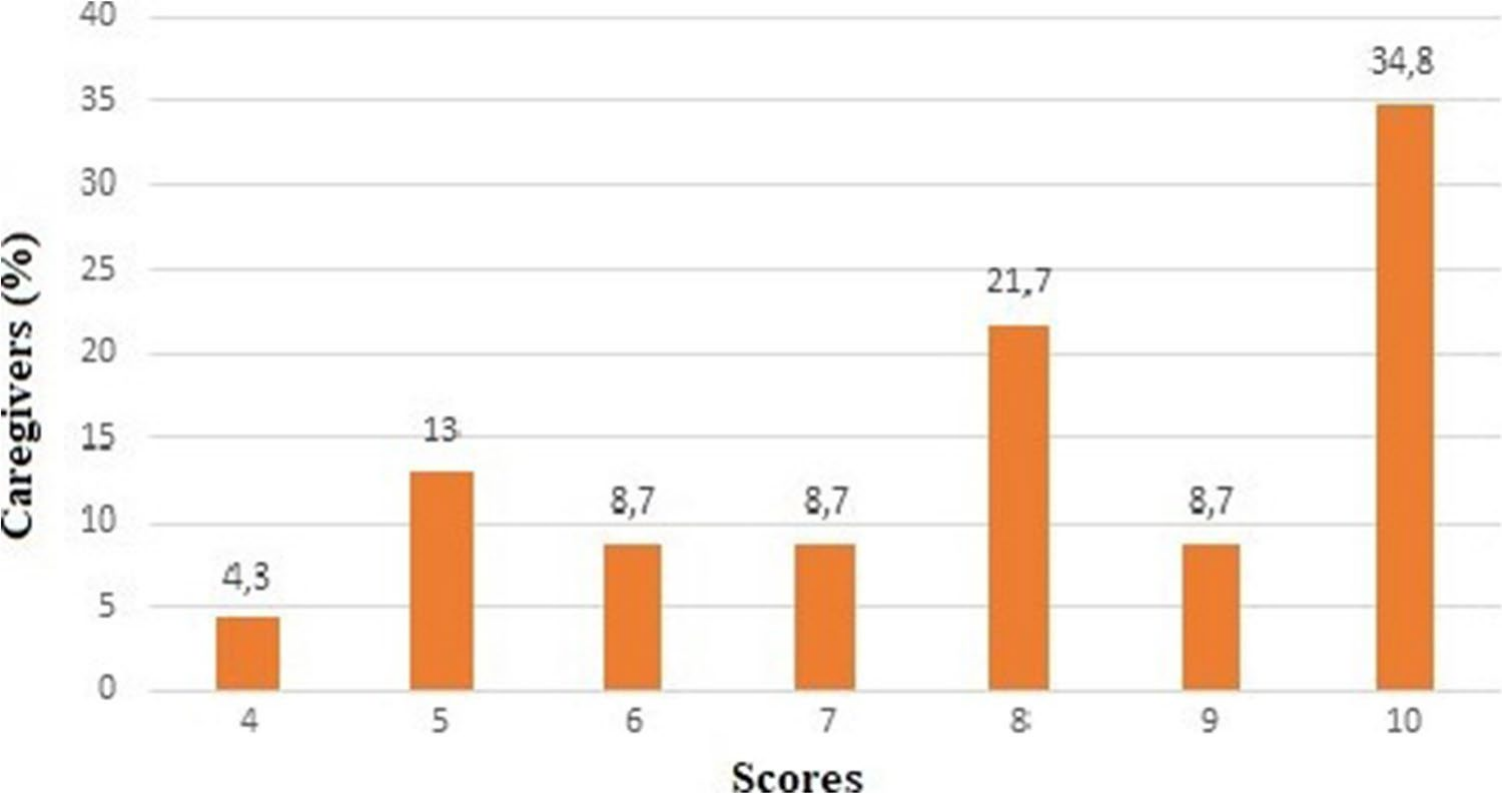
Satisfaction scores on a 1-to-10 scale given by caregivers (N = 23) to visit performed through TM (Q20), *No patients gave a score lower than 4 on a 1-to-10 scale. Telemedicine Satisfaction Survey is reported in Appendix 2

On a scale from 1 to 10, the overall level of satisfaction was high for both patients (8.5 ± 1.4) and their caregivers or family members (8.0 ± 2.0) (Q19-Q20) (Figs. 2 and 3).

## Discussion

In this study we investigated the comparability of neurological evaluations performed through TM compared with in-person visits in the management of patients with mild MS.

The in-person and remote inter-rater agreement was moderate in our analysis for the attribution of EDSS and most FS score. In the study population, κ coefficient was higher than 0.50 for pyramidal, brainstem, sensory, bowel and bladder FS scores, while lower values were reported for cerebellar, cerebral and visual FS scores. Similarly, in a study involving 41 participants with MS undergoing both in-person and TM evaluations, correlations between FS scores were modest for visual FS (r = 0.36), between 0.40 and 0.49 for sensory and cerebellar, between 0.50 and 0.60 for cerebral, pyramidal and brainstem FS scores [10].

Differently, the remote inter-rater agreement was substantial and κ coefficient was greater than 0.80 for all scores independently attributed by two neurologists. This is a consistent result, especially when considering the complex and the operator-dependent nature of the neurological examination, as confirmed by results from studies assessing the inter-rater agreement of the traditional in-person neurological examination[15–17]. Particularly, a moderate accordance for strength (κ = 0.54) and a good one for sensory evaluation (κ = 0.48 for pallesthesia, κ = 0.69 for sensibility to touch) of the upper limbs were reported in a study involving 41 patients with neurological disorders[16]. Another study reported a higher inter-rater agreement for observable neurological signs (κ = 0.70) than for elicitable ones (κ = 0.41), with a fair agreement for sensory evaluation and a substantial accordance for strength, gait and coordination [15].

Only visual FS score was significantly different between remote and in-person neurological examinations in our study, and particularly, it was underestimated during TM visit. However, when considering separately the inter-rater agreement in PwoC and PwC, accordance increased from poor to almost perfect. This could be easily explained, since the presence of caregivers allowed clinicians to properly assess the visual function by administering a pocket Snellen eye chart to patients, under the remote guidance of the neurologist. Differently, patients lacking of caregivers during the TM visit only reported subjective perceptions about any decrease in visual acuity or visual impairment. Moreover, among patients evaluated with the involvement of a caregiver, the inter-rater agreement was higher for all scores except for pyramidal FS score, which was not significantly different between groups.

There was a positive strong correlation between EDSS scores attributed during remote and face-to-face evaluations, in line with results reported by Bove and co-authors (r = 0.89) [10]. In this study, 30 out of 41 patients exhibited an EDSS 0.0–3.5, as in ours, and similar correlations were reported between tele-EDSS and FS scores and in-person ones. In the subset of 11 patients with EDSS 4.0–7.0, the authors reported even higher values of agreement between in-person and remote EDSS scores. Patients with EDSS scores higher than 3.5 were not recruited in our pilot study, since we decided to evaluate the role of caregivers’ support, which would be mandatory for more disabled patients, and to compare remote and in-person neurological examination under less demanding conditions. Moreover, higher EDSS scores mostly depend on ambulatory function, which cannot be adequately evaluated remotely.

In this regard, an ΔEDSS of 3.0 points was attributed during the remote visit to a patient reporting an ambulatory function restricted to 500 m due to severe fatigue, but objectively evaluated as an antalgic gait during in-person visit not affecting ambulation as previously reported. This limitation could be overcome with the use of specific wearable devices, able to evaluate gait, posture and balance [18–20]. Besides a passive monitoring, several smartphone applications are currently under evaluation, able to explore gait (2-min walk test, climbing stairs, balance), upper limb functions (finger-to-nose, handgrip, drawing), cognitive functions (Symbol Digit Modalities Test, memory tests), visual function [21, 22].

These tools may be a valid help in the assessment of both pyramidal and cerebellar FS scores, which reveals some difficulties during TM visit, especially when a caregiver is not available. In this case, particularly, safety issues have to be solved: In our study a patient fell on the ground during TM visit, without reporting any injuries, while executing the hop test in absence of reported strength or balance impairments. Probably, based on patient-reported outcomes, some tasks should not be performed, while others could be not easily performable if the setting is not appropriate, requiring the supine position (lower limbs position test, heel-to-shin test). This implies that the examination of the lower limbs may be not so accurate. Similarly, the examination of sensory functions could also be altered when a caregiver is not available and pallesthesia, proprioception and discrimination are difficult to assess through TM.

According to Moccia [23, 24], the highly multifaceted presentation of symptoms and sign in MS could represent a limitation in the use of TM, which could be employed to perform therapeutic consultations or to remotely guide caregivers and general practitioners at the bedside of severely disabled patients. Even more, clinical relapses could be particularly difficult to be excluded without a subsequent in-person evaluation or the performance of magnetic resonance imaging (MRI). In our study population, we only considered patients who did not experience clinical relapses or need steroid treatment in the previous month, with the purpose to explore the comparability between TM and in-person evaluations in the follow-up of patients with stable disease. Certainly, the detection of sign and symptoms suggestive of relapses with the use of TM can allow clinicians to identify patients benefiting from accessing the hospital setting. In this view, the overall accordance of remote and in-person evaluations was consistent in our sample and there is growing scientific evidence on the valid agreement between TM and in-person evaluations.

The presence of a caregiver, though assuring patients’ safety and improving the remote and in-person inter-rater agreement, can represent a limitation for patients with low disability, usually not requiring any assistance. Accordingly, the unavailability of a caregiver during TM visit led some patients to refuse the study entry. Beyond that, difficulties in using technologies and the unwillingness to be evaluated with TM were the main reason for refusal. On the other hand, when participants were asked to express their level of satisfaction with the use of TM to perform the follow-up neurological examinations, the overall level of satisfaction of both patients and caregivers was considerably high and only few patients declared their unwillingness to undergo further visits performed through TM. This agrees with results from other studies investigating the use of TM for patients with chronic diseases, confirming levels of satisfaction above 80% [25], although patients tend to consider in-person visits as more appropriate to discuss psychosocial and emotional issues[26–28].

In our sample, patients who accepted to undergo TM evaluations were substantially younger than those who refused and the percentage of subjects graduating from high school or having a degree was higher in the first group. However, results were not confirmed in multivariate analysis, possibly due to the small sample size. Previous studies reported an older age, a lower socioeconomic status, the male sex and lower levels of education as factors which negatively impact the use of e-health services [1, 29–31]. A certain distrust in the use of technology to substitute traditional evaluations is comprehensible, considering the recent and not yet widespread concept of e-Health and smart healthcare among the general population. However, this might limit the health access of part of the target population, especially older people, creating a “digital divide.” On the other hand, the availability of Internet at homes and cell phones, with access to web content anytime and anywhere, has already spread in most families and, in this perspective too, the role of the caregiver can be helpful.

Results from the administered patient’s satisfaction questionnaire confirmed that the TM visit was greatly appreciated by patients and caregivers and largely considered as emotionally involving, practical, accurate and easy to organize. Besides allowing time saving, 40% of respondents considered TM useful to save money. Actually, the use of TM could reduce both direct costs, particularly the outpatient-care ones, and indirect costs related to the management of MS, decreasing the loss of sick leaves and work days of absence [32].

This pilot study has some limitations. First, only patients with mild MS were included in this phase and the previous EDSS score was known to fall within the range 0.0–3.5, possibly influencing the attribution of EDSS score during the evaluations. However, this possible bias affected both remote and in-presence examiners, who were equally aware of the inclusion criteria. Further, such a condition usually occurs in clinical practice, since clinical data and reports of previous evaluations are stored in a computerized database and always available during outpatient visits. Further, subtle changes in the neurological examination could not be observed in a single assessment, needing a longer observation period and thus requiring a longitudinal study.

## Conclusion

This study supports the use of TM in the management of patients with MS. Participants declared a high level of satisfaction with the remote visit, and most of them were willing to consider the use of TM for forthcoming follow-up evaluations, although some of them expressed initial reluctance. Additionally, the use of TM has several advantages, allowing time and cost-saving and limiting access to the outpatient setting, with the added value of avoiding gatherings in the current context of COVID-19 pandemic [7].

In patients with low EDSS score and stable, the accuracy of the neurological examination in remote visits has proved to be acceptable for the attribution of EDSS and almost all FS scores. Further, the presence of a caregiver can allow physicians to perform a more complete and accurate examination in secure conditions. However, patient-reported gait worsening and visual disturbances probably require a following in-person evaluation. In this view, and even more in the context of COVID-19 pandemic, the use of TM could be worthwhile, allowing clinicians to distinguish patients who can benefit from accessing the hospital setting without taking unnecessary risks. Additionally, the complementary use of televisits and in-person evaluations in clinical practice may improve patients’ disease monitoring without excessively penalize the doctor–patient relationship. Further studies could clarify the potentialities of TM in more disabled MS patients and enforce the use of wearable devices to overcome TM limitations.

## Supplementary Information

Below is the link to the electronic supplementary material.

Supplementary file1 (PDF 91 KB)

Supplementary file2 (PDF 106 KB)

## Data Availability

The data that support the findings of this study are available from the corresponding author upon reasonable request. **Ethical Statement.** The study was approved by our local ethical committee and was performed in accordance with the ethical standards as laid down in the 1964 Declaration of Helsinki and its later amendments or comparable ethical standards. All patients provided signed written informed consent accepting to participate the study and publication.
