# Supplementary material for: Reliability of televisits for patients with mild relapsing–remitting multiple sclerosis in the COVID-19 era
Source: Neurol Sci. 2022 Jan 11;43(4):2253–61. doi: 10.1007/s10072-022-05868-5 (PMC8751468; doi:10.1007/s10072-022-05868-5)
Supplement: Supplementary file 1 — Supplementary file1 (PDF 91 KB) [file 10072_2022_5868_MOESM1_ESM.pdf]

Reliability of Televisits for patients with mild relapsing-remitting Multiple Sclerosis in the COVID-19 era

Neurological Sciences

Toscano S[1]*, Patti F[1]*, Chisari CG[1], Arena S[1], Finocchiaro C[1], Schillaci CE[2], Zappia M[1]

[1]Department "G.F. Ingrassia", Section of Neurosciences, Neurology Clinic, University of Catania, 95123 Catania, Italy

[2]Department of Economics and Business, University of Catania, 95129 Catania, Italy

Corresponding author: Prof. Francesco Patti; patti@unict.it

Model for neurological examination performed in Telemedicine

1. Collection of anamnestic data and report of the patient's perception about his/her disease course, as follows:
    - Patient's perception of disease course:
        a) Stable disease
        b) Worsening condition
        c) Improved condition
    - Ongoing treatment with DMDs, evaluation of compliance and side effects
    - Concomitant treatment
    - Laboratory findings and MRI, previously evaluated by the physician after being sent by the patient to a repository software

2. Ambulation: patient's report about maximal unassisted walking distance, type of assistance required, eventual gait impairment.

3. Neurological exam and attribution of FS and EDSS scores, as follows:

Pyramidal function:

Firstly, the examiner asks the patient to refer any impairment in muscle strength and balance. Thus, the examiner decides whether the following tasks can be safely performed (also considering the presence/absence of a caregiver):

a) Pronation and downward drift: the patient will be asked to stretch his/her arms in front, hands in the same horizontal plane, the fingers spread and the eyes closed. The examiner observes downward drift of the hand after one minute.
b) Hopping: the examiner asks the patient to stand and hop on one foot more than 15 times, then the test is repeated with the other leg.

Cerebellar function:

Firstly, the examiner asks the patient to refer any impairment in both static and dynamic balance. Thus, the examiner decides whether the following tasks can be safely performed (also considering the presence/absence of a caregiver):

a) Finger-to-nose test: the patient touches the tip of his/her nose with the index finger of each hand, repeating the test both with open and closed eyes. The test will be repeated several times with each hand.
b) Romberg test: the patient is asked to stand with feet together, eyes open and hands by the sides or arms outstretched, looking at the ceiling. Then the subject closes the eyes while the examiner observes for a full minute.

<u>Brainstem function:</u>

a) Extraocular movements impairment (III, IV, VI cranial nerves, c.n.): the patient is asked to sit with his/her head up and looking straight ahead. The examiner moves a pen (or other comparable object) in several directions asking the patient to follow it with eyes, without moving the head. The occurrence of nystagmus will also be evaluated.
b) Sensation on the face and muscles of mastication (V c.n.): superficial sensation in the three trigeminal branches will be evaluated by the caregiver with his fingers, if available, or by the patient himself/herself. Corresponding areas on both sides of the face will be assessed. The patient is also asked to clench his/her teeth, to chew and to open the mouth.
c) Facial motor function (VII c.n.): the examiner inspects the face of the patient, looking for asymmetries. The patient is asked to wrinkle his/her forehead or raise the eyebrows, to tightly close the eyes, to puff out both cheeks and show his/her teeth.
d) Hearing and vestibular disorders (VIII c.n.): the patient is asked about hearing loss, vertigo and balance disorders.
e) Phonation: the patient is asked to pronounce out loud the letter A, then to read a short text, easily accessible in his/her setting.
f) Swallowing: the patient is asked about any difficulties in swallowing with both liquid and solid foods, the need for a diet modified in consistency, the occurrence of coughing at mealtime and the eventual increase in time taken to complete the meal.
g) Trapezius and sternocleidomastoid muscles (XI c.n.): the patient will be asked to rotate his/her head to both the left and the right sides, then to bend the neck forward.
h) Tongue motility (XII c.n.): the patient is asked to open his/her mouth and to protrude the tongue, then moving it to both sides.

<u>Sensory function</u>:

Whether a caregiver is available, he/she will be guided by the examiner to perform the tasks. Otherwise, the patient himself/herself will be guided to perform the tasks.

a) Superficial sensation in upper limbs: the caregiver/the patient is asked to touch corresponding areas on both arms with his/her fingers, reporting any differences in sensation.
b) Superficial sensation in lower limbs: the caregiver/the patient is asked to touch corresponding areas on both legs with his/her fingers, reporting any differences in sensation.

c) Lhermitte’s sign: the patient is asked to bend his/her head forward, in order to elicit the occurrence of a sudden sensation resembling an electric shock passing down the back of the neck and into the spine, possibly radiating out into arms and legs.

Visual function:

The examiner investigates about any decrease in visual acuity, visual impairments, scotomas and visual field defects. Whether a caregiver is available, he/she will be provided with a printable pocket Snellen Eye chart (18.5 cm x 10 cm) and instructions for use (the chart will be placed 6 feet away from the patient).

Bowel and bladder functions:

The FS score will be determined according to the related section of the Neurostatus scoring [30].

The examiner investigates about any impairments in bladder function, particularly:

- urinary hesitancy/retention;
- urinary urgency/incontinence;
- necessity to use urinals, pads, intermittent catheterisation or continuous bladder drainage;
- impact on quality of life.

The examiner also investigates about any impairments in bowel function, particularly:

- incontinence/constipation;
- necessity to use pads or manual measures to evacuate;
- impact on quality of life.

The examiner investigates about any impairments in sexual function, particularly:

- for males: difficulty to achieve and/or maintain erection during intercourse, decrease in libido, hypo-orgasmia
- for females: lack of lubrication, dyspareunia, hypo-orgasmia, decrease in sexual activity

Cerebral functions:

The occurrence of mood disorders, fatigue and decrease in mentation are investigated.
