# Supplementary material for: Reliability of televisits for patients with mild relapsing–remitting multiple sclerosis in the COVID-19 era
Source: Neurol Sci. 2022 Jan 11;43(4):2253–61. doi: 10.1007/s10072-022-05868-5 (PMC8751468; doi:10.1007/s10072-022-05868-5)
Supplement: Supplementary file 2 — Supplementary file2 (PDF 106 KB) [file 10072_2022_5868_MOESM2_ESM.pdf]

Reliability of Televisits for patients with mild relapsing-remitting Multiple Sclerosis in the COVID-19 era

Neurological Sciences

Toscano S[1]*, Patti F[1]*, Chisari CG[1], Arena S[1], Finocchiaro C[1], Schillaci CE[2], Zappia M[1]

[1]Department "G.F. Ingrassia", Section of Neurosciences, Neurology Clinic, University of Catania, 95123 Catania, Italy

[2]Department of Economics and Business, University of Catania, 95129 Catania, Italy

Corresponding author: Prof. Francesco Patti; patti@unict.it

Patient's satisfaction questionnaire about TM evaluation

1. È riuscito a comunicare al medico ciò che desiderava dirgli?

   Sì

   No

2. Le sembra di aver capito tutto ciò che il medico intendeva dirle?

   Sì

   No

3. Da 1 a 10, ritiene di essere stato sottoposto ad una visita accurata in relazione alla sua patologia?

   1=per nulla; 10=moltissimo

4. Ha incontrato difficoltà nella effettuazione della Televisita?

   Sì

   No

5. Se sì, quante?

   Poche

   Abbastanza

   Molte

6. Se sì, in che momento?

Attivare il collegamento

Qualità della connessione

Svolgimento del colloquio

Svolgimento della visita neurologica

7. Ha avuto difficoltà tecniche a:

   Sentire il medico

   Vedere il medico

   Farsi sentire dal medico

   Farsi vedere dal medico

8. Ci sono stati ritardi tra voce e video?

   Sì

   No

9. La connessione si è interrotta durante la Televisita?

   Sì

   No

10. L’orario che avete fissato è stato rispettato?

    Sì

    No

11. Pensa che la Televisita abbia determinato un risparmio economico?

    Sì

    No

12. Da 1 a 10, quanto ritiene sia stato complicato a livello organizzativo effettuare questa Televisita?

    1=per nulla; 10=moltissimo

13. Da 1 a 10, quanto ritiene sia stata fredda e distaccata questa Televisita?

1=per nulla; 10=moltissimo

14. Da 1 a 10, quanto ritiene sia stata coinvolgente questa Televisita?

    1=per nulla; 10=moltissimo

15. Da 1 a 10, quanto ritiene sia stata comoda questa Televisita?

    1=per nulla; 10=moltissimo

16. È stato difficile trovare uno spazio per effettuare la Televisita?

    Sì

    No

17. Lo spazio in cui ha effettuato la Televisita era adeguato allo scopo?

    Sì

    No

18. Sulla base di questa esperienza, eseguirebbe in Teleconsulto anche il prossimo controllo ambulatoriale?

    Sì

    No

19. Da 1 a 10, quanto si ritiene soddisfatto della Televisita effettuata?

    1=per nulla; 10=moltissimo

20. Per i familiari con cui vive o per le persone che la assistono direttamente (caregivers), se presenti alla visita: da 1 a 10, quanto ritengono soddisfacente la Televisita che ha effettuato?

    1=per nulla; 10=moltissimo

Adapted from "Teleconsultation patients' questionnaire" freely available at the website https://www.istituto-besta.it/televisita/questionario [11].
